# Evaluation of Resistance-Associated Substitutions in NS5A Using Direct Sequence and Cycleave Method and Treatment Outcome with Daclatasvir and Asunaprevir for Chronic Hepatitis C Genotype 1

**DOI:** 10.1371/journal.pone.0163884

**Published:** 2016-09-29

**Authors:** Tatsuya Ide, Yuichiro Eguchi, Masaru Harada, Kunihide Ishii, Masaru Morita, Yasuyo Morita, Gen Sugiyama, Hirofumi Fukushima, Yoichi Yano, Kazunori Noguchi, Hiroki Nakamura, Junjiro Hisatomi, Hiroto Kumemura, Miki Shirachi, Shinji Iwane, Michiaki Okada, Yuichi Honma, Teruko Arinaga-Hino, Ichiro Miyajima, Kei Ogata, Reiichiro Kuwahara, Keisuke Amano, Toshihiro Kawaguchi, Ryoko Kuromatsu, Takuji Torimura, DAAs Multicenter Study Group

**Affiliations:** 1 Division of Gastroenterology, Department of Medicine, Kurume University School of Medicine, Fukuoka, Japan; 2 Department of Internal Medicine, Saga Medical School, Saga, Japan; 3 Third Department of Internal Medicine, University of Occupational and Environmental Health, Fukuoka, Japan; 4 Asakura Medical Association Hospital, Fukuoka, Japan; 5 Yame General Hospital, Fukuoka, Japan; 6 Nagata Hospital, Fukuoka, Japan; 7 Kurume University Medical Center, Fukuoka, Japan; 8 Saiseikai Futsukaichi Hospital, Fukuoka, Japan; 9 Saga Central Hospital, Saga, Japan; 10 Omuta City Hospital, Fukuoka, Japan; 11 Shin Koga Clinic, Fukuoka, Japan; 12 Kurume Chuo Hosipital, Fukuoka, Japan; 13 Kumamoto Central Hospital, Kumamoto, Japan; 14 Chikugo City Hospital, Fukuoka, Japan; National Taiwan University Hospital, TAIWAN

## Abstract

**Background:**

The aim of this study was to evaluate the efficacy of daclatasvir plus asunaprevir therapy in patients infected with hepatitis C virus and determine its relevance to resistant variants.

**Methods:**

A total of 629 consecutive patients infected with hepatitis C virus genotype 1 were assessed. Daclatasvir (60 mg/day) plus asunaprevir (200 mg/day) was given for 24 weeks. The virological responses and resistance-associated substitutions of hepatitis C virus mutants were examined by the direct sequence and cycleave methods were evaluated.

**Results:**

Overall, 89.4% (555/621) of patients exhibited a sustained virological response (SVR). The SVR rates in the patients with wild type, mixed, and mutant type Y93 by direct sequencing were 92.5% (520/562), 70.3% (26/37), and 42.9% (9/21), respectively. The SVR rates in the patients with 100%, 90%, 80%-30%, and 20%-0% Y93 wild by the cycleave method were 93.4% (456/488), 88.2%(30/34), 56.0%(14/25), and 36.8%(7/19), respectively. In contrast, the SVR rates for the wild type and mixed/mutant type L31 by direct sequencing were 90.2% (534/592) and 72.4% (21/29), respectively. In the multivariate analyses, the wild type Y93, no history of simeprevir therapy, the wild type L31, and low HCV RNA level were independent factors of SVR.

**Conclusion:**

NS5A resistance-associated substitutions, especially Y93H, were major factors predicting the SVR. Although direct sequencing can predict the SVR rate, the cycleave method is considered to be more useful for predicting the SVR when used in combination.

## Introduction

Hepatitis C virus (HCV) is the leading cause of liver-related death worldwide [1]. For a decade, the standard of care for treatment of chronic hepatitis C was peg-interferon and ribavirin-based regimens. However, these treatments have significant side effects, suboptimal rates of sustained virologic response (SVR), and a long treatment duration [2–4].

Recently, the treatment of HCV infection has made significant advances with the development of new direct-acting antivirals [5–8]. In Japan, combination therapy of asunaprevir and daclatasvir was approved in September 2014 as the first interferon-free regimen for genotype 1. Although high rates (87.4%) of SVR were obtained in Japanese clinical trials, the SVR rates in patients with resistant variants (L31M/V and/or Y93H) of NS5A were low (40.5%) [9]. Identifying the patients with such resistant variants is important for tailoring therapies. While a direct sequencing analysis is generally used to detect resistance-associated substitutions (RASs), new methods of detecting RASs have been developed.

A novel simple assay for quantifying the percent of NS5A Y93H mutant and Y93 wild-type strain HCV-RNA relative to the total HCV-RNA has been reported by Uchida et al [10]. They also revealed the characteristics of patients with Y93 mutation using this method [11]. This method has come to be used often in Japan. However, the details regarding the relationship between the SVR rate and the proportion of resistant variants are unclear. In addition, given that this therapy has some side effects, the prediction of SVR before treatment is very important.

For these reasons, the authors conducted a prospective multicenter asunaprevir and daclatasvir treatment study and investigated the proportions of resistant variants and the viral response to predict the SVR rates.

## Patients and Methods

In this multicenter study, 629 consecutive patients who received the asunaprevir and daclatasvir combination therapy between September 2014 and January 2015 were enrolled. All of the patients had chronic HCV genotype (serotype) 1 infection. Compensated cirrhosis patients (Child-Pugh A) as well as chronic hepatitis were enrolled. The attending physician clinically diagnosed the presence of cirrhosis. Asunaprevir was administered orally at a dose of 100 mg twice daily, and daclatasvir was administered orally at a dose of 60 mg once daily, both for 24 weeks. The laboratory tests for evaluating the liver function were conducted every two weeks.

### Measurements of serum HCV RNA levels and RASs

HCV RNA levels were measured using the Cobas AmpliPrep/Cobas TaqMan HCV test, version 2.0 (Roche Diagnostics, Tokyo, Japan). The TaqMan has a lower limit of quantification of 1.2 log IU/mL and an upper limit of quantification of 7.8 log IU/mL [12,13]. Below the lower limit of quantification, HCV RNA is said to be “unquantifiable” and is further qualified as either target detected or target not detected. A response of ‘target not detected’ is defined as HCV RNA-negative.

HCV RNA levels were measured at baseline; weeks 2, 4, 8, 12, 16, 20, and 24; and post-treatment at weeks 4 and 12. Direct sequencing and the cycleave method of the NS5A gene were performed at baseline. The L31 (M or V or F) and Y93 (H or F or C or R) mutations were detected by direct sequencing, and the Y93H mutant strain was measured by the cycleave method [10] by SRL, Inc. (Tokyo, Japan). The method and primer sets are the same as those used by Uchida et al [10].

Direct sequencing of Y93 revealed the wild type (Y93Y), mixed type (Y93Y/H), and mutant type (Y93H). Direct sequencing of L31 revealed the wild type (L31L), mixed type (L31L/M/V), and mutant type (L31M/V). The results of the cycleave method revealed the percentages of Y93 wild strains and Y93H mutant strains, presented as a ratio; for example, if the percentage of Y93 wild strains was 90% and that of Y93H mutant strains was 10%, the result was expressed as 90/10.

### End points

A SVR was defined as being HCV RNA negative in the serum at 12 weeks after the end of therapy. Virologic breakthrough (VBT) was defined as a detectable HCV RNA level at any time after the HCV RNA level had been negative during the treatment. A null virologic response (NVR) was defined as a sustained detectable HCV RNA level during the treatment. Virologic relapse was defined as a detectable HCV RNA level during the 12-week post-treatment period in patients who had negative HCV RNA at the end of treatment. A rapid virological response (RVR) was defined as negative HCV RNA at Week 4 of therapy. The study protocol was in compliance with the Helsinki Declaration and was approved by the ethics committee of each participating institution (Ethical Committee of Human Experimentation in Kurume University School of Medicine (approval No. 14178), Human and Animal Ethics Review Committee in University of Occupational and Environmental Health (approval No.26-111), Ethics committee of Saga University (IRB No.2015-09-06)). Written informed consent was obtained from all the patients.

### Statistical analyses

The associations between the L31 and Y93 types and the HCV RNA negative rate were analyzed using Fisher’s exact test. The baseline factors influencing the probability of an SVR were examined using univariate and multivariate logistic regression analyses. All of the analyses were performed using the JMP pro 11 software program (SAS Institute, Cary, NC, USA).

## Results

### Patients

**Fig 1:**
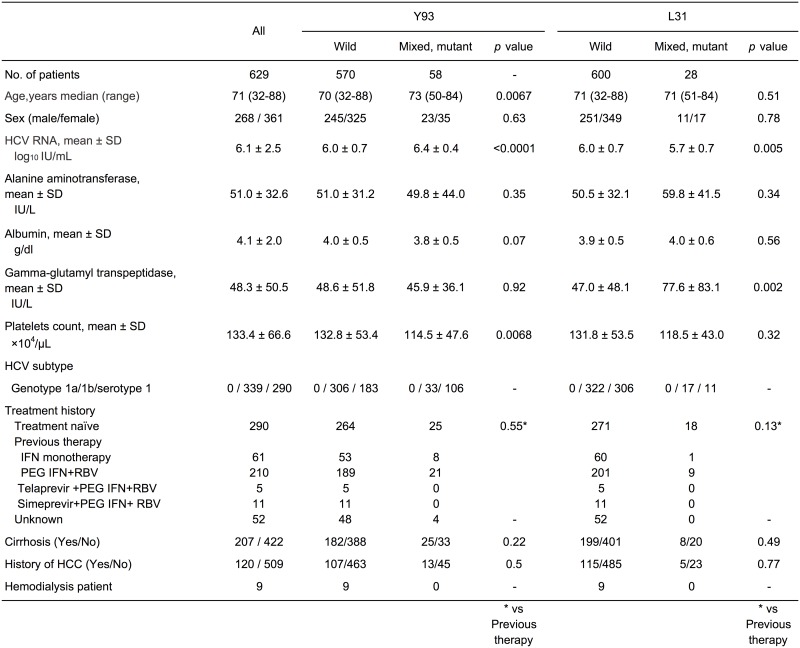
Baseline characteristics.

Fig 1 summarizes the patient characteristics. The average age was relatively high, and the patients more than 70 years old accounted for 55.3% (348/629) of the total population. The patients who had Y93 wild type tended to be younger and have lower HCV RNA titers and higher platelets counts than those with Y93 mixed and mutant type. The patients who had L31 wild type tended to have higher HCV RNA titers and lower gamma-GTP levels than those with Y93 mixed and mutant type. There was no relationship between previous treatment and Y93/L31 mutation status.

Table 1 shows the number of patients with L31 and Y93 mutations as determined by direct sequencing. Y93 mutants (mixed, mutant type, and other mutant) were detected in 58 patients (9.2%, 58/629), and L31 mutants (mixed, mutant type, and L31F) were detected in 28 patients (4.5%, 28/629). In addition, both L31 and Y93 mutants were detected in two patients (Y93 mixed and L31 mixed, and Y93 mixed and L31 mutant, one each).

**Table 1:** The L31 and Y93 mutation distribution by direct sequencing.

		L31				
	Type	Wild	Mixed	Mutant	Other mutant	total
Y93	Wild	544	5	20	L31F(n = 1)	570
	Mixed	35	1	1	0	37
	Mutant	20	0	0	0	20
	Other mutant	Y93F(n = 1)	0	0	not detected(n = 1)	2
	total	600	6	21	2	629

### Virologic response

**Fig 2:**
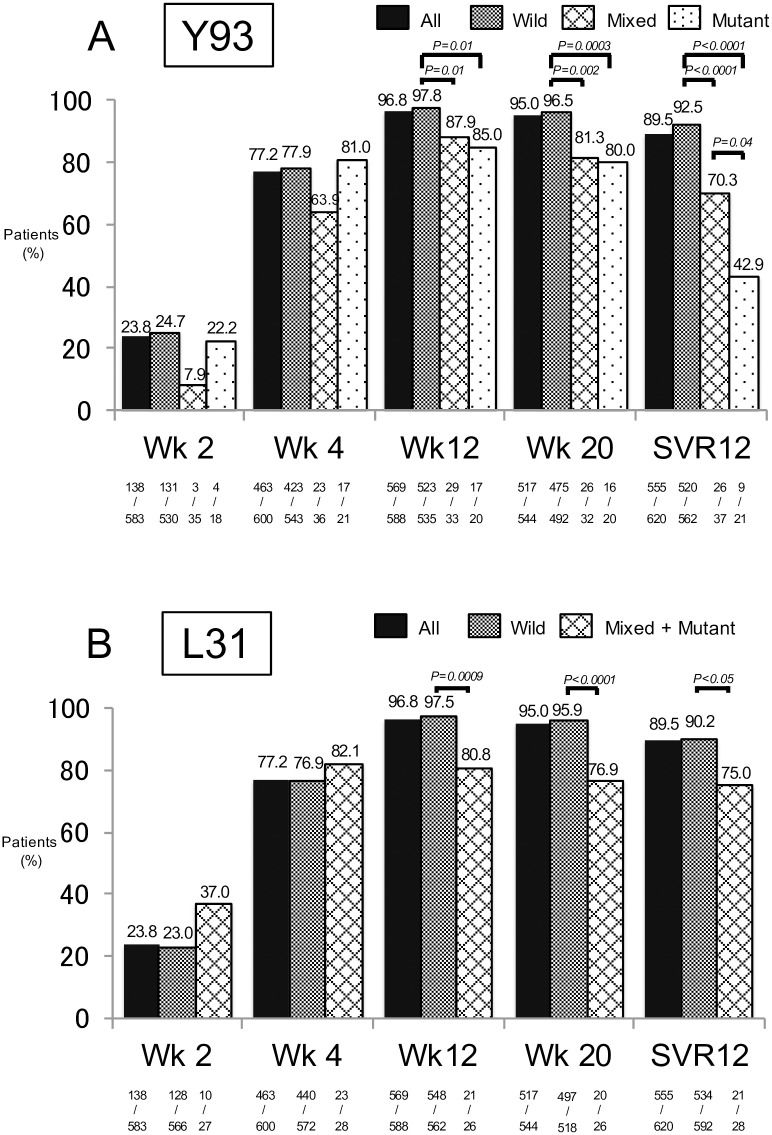
The virological response during and after treatment according to Y93 and L31 baseline status by direct sequencing. (A) Y93. (B) L31. One patient for whom Y93 and L31 results were not obtained was excluded. Five patients who were lost to follow-up and three patients who died were not included. The HCV RNA negative rate was analyzed using Fisher’s exact test.

Overall, since 8 of the 629 initial patients were lost to follow-up, 621 patients were evaluated for an SVR. The SVR rate was 89.4% (555/621). The percentage of patients with HCV RNA negative during and 12 weeks after treatment (SVR12) are shown in Fig 2A and 2B for the Y93 and L31 type, respectively, examined by direct sequencing. Since few patients had the L31 mixed type (n = 6), L31 mixed and mutant types were combined. Since 1 patient was not detected using direct sequencing, 620 patients were investigated in Fig 2. The response rates were higher for the Y93 wild type during treatment, and there was a significant difference in the percentages at 12 and 20 weeks of treatment and 12 weeks after treatment. The lowest SVR12 was observed for the Y93 mutant type. In contrast, the response rates were significantly higher for the L31 wild type than for the mixed and mutant types at 12 and 20 weeks of treatment and 12 weeks after treatment. Among the 58 non-SVR patients, except for those who discontinued the treatment due to adverse events, virologic relapse and VBT accounted for 44.8% (n = 26), and NVR accounted for 10.3% (n = 6). One patient for whom Y93 and L31 results were not obtained achieved SVR.

**Fig 3:**
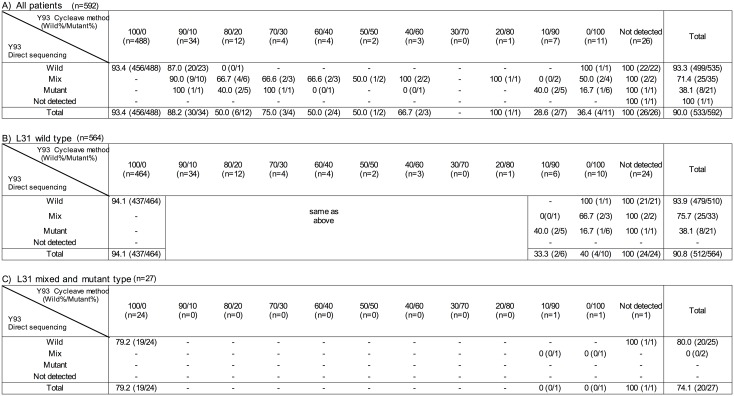
The sustained virological response rates according to Y93 mutation status by direct sequencing and the cycleave method. Panel A, all patients; Panel B, patients with L31 wild type; Panel C, patients with L31 mixed and mutant types. The L31 result was not obtained in one patient.

We analyzed the SVR rate and number of patients according to Y93 mutation status by direct sequencing and the cycleave method (Fig 3) The 37 patients who discontinued the treatment within 16 weeks or were lost to follow-up were excluded in order to investigate the precise relationship between the treatment efficacy and mutation status. Fig 3A shows the findings for all patients, Fig 3B shows the findings for the patients who had L31 wild type, and Fig 3C shows the findings for the patients who had L31 mixed and mutant types. The highest SVR rate (94.1%) was observed in the patients who had wild type L31 and Y93 by direct sequencing and 100% wild type by the cycleave method. Two patients had both L31 and Y93 mixed or mutant, and neither achieved SVR. Of the 25 patients who discontinued the treatment due to elevated levels of ALT from 2 to 24 weeks, 23 (92.0%) achieved SVR. The median treatment duration of these SVR patients was 12 weeks.

### Factors associated with SVR

The factors associated with SVR are shown in Table 2. According to the univariate analysis, the following factors were associated with SVR: low HCV RNA level, no history of simeprevir treatment, L31 and Y93 wild type. The same four factors were identified by multivariate analysis as independent factors that significantly influenced on SVR. We also analyzed the SVR rate according to these different parameters (Table 3). The highest SVR rate (96.8%) was observed in the patients who had wild type Y93 and L31 by direct sequencing, 100% wild type by the cycleave method, <6.1 log_10_IU/ml of HCV RNA and no simeprevir treatment history.

**Table 2:** The factors associated with a sustained virological response (univariate and multivariate analyses).

	Univariate			Multivariate		
	Odds ratio	95% CI	*p* value	Odds ratio	95% CI	*p* value
Sex: male	1.21	0.73–2.03	0.454	0.96	0.52–1.78	0.899
Age	0.99	0.96–1.02	0.628	1.00	0.97–1.04	0.877
HCV RNA level	0.47	0.29–0.73	0.0006	0.46	0.26–0.77	0.002
Alanine aminotransferase	0.99	0.99–1.00	0.444	0.99	0.99–1.01	0.91
Albumin	0.69	0.38–1.22	0.211	1.00	0.85–1.72	0.992
Gamma-glutamyl transpeptidase	0.99	0.99–1.00	0.208	0.99	0.99–1.00	0.363
Platelet count	1.03	0.98–1.08	0.268	1.01	0.94–1.08	0.794
Cirrhosis: No	1.01	0.58–1.72	0.961	0.92	0.44–1.78	0.817
Simeprevir treatment history: no	16.3	4.8–63.9	P<0.0001	44.4	10.7–223	P<0.0001
L31: wild type	3.01	1.14–7.07	0.027	5.08	1.70–13.9	0.005
Y93: wild type	8.74	4.72–16.1	P<0.0001	8.57	4.23–17.3	P<0.0001

**Table 3:** SVR rates for the different parameters.

Y93		L31	HCV RNA (Log_10_ IU/ml)	Simeprevir treatment history	SVR rate
Direct sequencing	Cycleave Method (Wild%/Mutant%)	Direct sequencing			
Wild	100/0	Wild	≤ 6.1	No	96.8 (239/247)
Wild	100/0	Wild	≥ 6.2	No	94.1 (190/202)
Wild	100/0	Wild	All	Yes	36.4 (4/11)
Wild	90/10	Wild	≤ 6.1	No	71.4 (5/7)
Wild	90/10	Wild	≥ 6.2	No	93.8 (15/16)
Mixed	90/10	Wild	All	No	90.0 (9/10)
Mixed	80/10–40/60	Wild	All	No	68.5 (11/16)
Mixed	10/90–0/100	Wild	All	No	50.0 (2/4)
Wild	100/0	Mixed, mutant	≤ 6.1	No	82.4 (14/17)
Wild	100/0	Mixed, mutant	≥ 6.2	No	71.4 (5/7)

The baseline HCV RNA level for an SVR was 6.0±0.7 LogIU/ml and that for non-SVR was 6.3±0.5 LogIU/ml. Of the 11 patients who had a history of simeprevir/peginterferon/ribavirin treatment, 7 (63.6%) did not achieve SVR. All 11 patients were Y93 and L31 wild type by direct sequencing and 100% wild type by the cycleave method. On excluding these 11 patients from Fig 3B, the SVR rate of the patients who had Y93 and L31 wild type by direct sequencing and 100% wild type by the cycleave method was 94.9% (430/453). One patient who had a history of telaprevir/peginterferon/ribavirin treatment achieved an SVR. We evaluated the Y93H mutation by direct sequencing in 14 non SVR patients after 6 month of the therapy, 12 patients (85.7%) had Y93H mutation.

### Safety

Of the 629 patients, 51 (8.1%) discontinued the treatment due to adverse events. Twenty-five patients discontinued due to elevated levels of ALT, 7 due to drug eruption, and 5 patients due to a fever. As for the other reasons for discontinuation, liver cancer (n = 3), transient consciousness loss (n = 1), increased total bilirubin level (n = 1), malaise (n = 1), stomatitis (n = 1), numb hands and feet (n = 1), headache (n = 1), patient’s wish (n = 2). Three patients died during the treatment: 2 drowned in the bathtub at Weeks 17 and 21, and 1 died of a cerebral hemorrhage of cavernous malformation at Week 8. Since these three patients had no other side effects during the therapy, we concluded that there was no relationship between the treatment and their deaths. Nine patients were on hemodialysis, and all of them completed the treatment safely and achieved an SVR.

## Discussion

This study evaluated 24-week treatment of daclatasvir plus asunaprevir in patients infected with HCV genotype 1. We affirmed the high efficacy of this therapy, and the average SVR rate was equivalent to that observed in a Japanese phase 3 clinical trial [9]. The SVR rate of patients with a Y93 mutation was low in the previous report [9], and we further demonstrated that the patients who had Y93 wild type achieved higher SVR rates than those who had Y93 mixed and mutant types. However, thus far, the degree to which the Y93H mutation influenced on the SVR rate has been unclear.

Recently, the cycleave method was developed to measure the proportion of Y93H mutations and is used widely in Japan. In this study, we evaluated the Y93 mutation by direct sequencing and the proportion of Y93H mutation by the cycleave method in all patients; the results were obtained in 99.8% (628/629) by direct sequencing and 95.6% (566/592) by the cycleave method. We were unable to obtain a result via direct sequencing in one patient, possibly because the patient had a low HCV RNA titer (2.9 LogIU/ml). However, the reason why no results were obtained by the cycleave method in 4.4% patients is unknown. Two reasons may explain the lower detection rate with cycleave method than with direct sequencing. One is that the cycling-probe did not hybridize the target portion in the cycleave method; the other reason is that the nested PCR was used in the direct sequencing.

In general, the rate of patients with a Y93 mutation based on a direct sequencing analysis is about 11%-23% [14,15]. In this study, the rate was relatively low (9.2%), as the patients who had mutations might have tended to avoid the treatment. However, we treated patients who had NS5A RASs if they were older and had advanced fibrosis or contraindications for interferon-based therapy. During the study period, we were only able to use daclatasvir plus asunaprevir therapy, as the sofosbuvir plus ledipasvir regimen, which is the second-generation all-oral regimen in Japan, was not approved until September 2015.

In addition, we demonstrated the SVR rate according to the proportion of Y93H mutations by the cycleave method. Since no precise SVR rate has been reported according to the mutation rate, our study is the first useful result for predicting the SVR rate of daclatasvir plus asunaprevir therapy using mutation rate.

Although we obtained similar SVR rates for Y93 via the direct sequence and cycleave method, the rates were still slightly different. For example, the SVR rate of the patients who had Y93 wild type (direct sequencing) and 100% wild (cycleave) was 93.4%; however, that of the patients who had Y93 wild type and 90% wild was 87.0%. In addition, the SVR rate of the patients who had Y93 mutant type (direct sequencing) was 38.1%, while that of those with Y93 mutant type and 0% wild (cycleave) was 16.7%. Therefore, we believe that the cycleave method can more accurately predict the SVR rate when used in combination with direct sequencing.

On the other hand, 32 patients who had no Y93 mutations did not achieve an SVR (Fig 3). In these patients, 5 had the L31 mutation, and 7 had a history of simeprevir treatment. NS3 RASs of D168 are known to develop after simeprevir failure [16]. Because simeprevir and asunaprevir are similar NS3 protease inhibitors, asunaprevir plus daclatasvir therapy is generally not effective in patients with simeprevir failure. Furthermore, there were 20 non-SVR patients who had no Y93 mutation, no L31 mutation, and no history of simeprevir therapy; the reasons for their failure to achieve an SVR were unclear but may have been due to variations in the asunaprevir concentration between patients [17], a very low concentration of resistant variants, [18] or other resistant variants. Further studies are needed to clarify this point in full. In our multivariate analysis, the Y93 and L31 wild type, no history of simeprevir treatment, and a low baseline HCV RNA level were also independent significant factors for SVR.

Recently, sofosbuvir and ledipasvir treatment was approved in Japan [19], and the SVR rate with this treatment is high, even in patients with Y93 mutations. However, patients with renal failure/hemodialysis [20] or arrhythmia treated with amiodaron are contraindicated for sofosbuvir-based regimens [21,22]. Therefore, these patients should be treated with non-sofosbuvir-including regimens. In hemodialysis patients, daclatasvir plus asunaprevir therapy was well tolerated and highly effective [23,24]. We also found that all hemodialysis patients achieved SVR in this study.

In the patients in whom daclatasvir and asunaprevir therapy fails to eradicate HCV, NS3 and/or HCV NS5A-inhibitor resistant variants emerge frequently after therapy [9]. Since the resistant variants—especially NS5A inhibitor-resistant virus—tend to persist for long time [8], the SVR rate will be limited in NS5A inhibitor combination regimens. As such, a new strategy for treating these patients should be designed in the future.

In conclusion, NS5A RASs are very important for predicting the SVR in daclatasvir and asunaprevir therapy, and the cycleave method is more useful for predicting the SVR rate when used in combination.
